# Multimodal Quantitative Parameter Mapping Characterizes Low-dimensional Feature Organization and Tract-specific Signatures in White Matter

**DOI:** 10.2463/mrms.mp.2026-0105

**Published:** 2026-07-28

**Authors:** Emi Sato, Yuki Kanazawa, Masafumi Harada, Yo Taniguchi, Masahiro Takizawa, Hiroaki Hayashi, Motoharu Sasaki, Akihiro Haga

**Affiliations:** 1 Graduate School of Health Sciences, Kumamoto University, Kumamoto, Kumamoto, Japan; 2 Department of Medical Imaging Physics, Faculty of Life Sciences, Kumamoto University, Kumamoto, Kumamoto, Japan; 3 Institute of Biomedical Sciences, Tokushima University Graduate School, Tokushima, Tokushima, Japan; 4 FUJIFILM Corporation, Tokyo, Japan; 5 Faculty of Transdisciplinary Sciences for Innovation, Kanazawa University, Kanazawa, Ishikawa, Japan

**Keywords:** diffusion kurtosis imaging, principal component analysis, quantitative magnetic resonance imaging, quantitative susceptibility mapping, white matter

## Abstract

**Purpose:**

To investigate relationships among relaxation, susceptibility, and diffusion parameters in healthy white matter (WM) and to characterize WM MRI feature organization using an integrative multimodal quantitative MRI framework.

**Methods:**

Twenty-two healthy volunteers underwent 3T MRI. Quantitative parameter mapping provided R_1_, R_2_*, R_1_·R_2_*, and quantitative susceptibility mapping, while diffusion kurtosis imaging provided fractional anisotropy (FA), mean kurtosis, axial kurtosis (AK), and radial kurtosis. All maps were spatially normalized to Montreal Neurological Institute (MNI) space, and mean values were extracted from WM tracts defined by the Johns Hopkins University (JHU) White Matter Atlas. Pearson correlation analysis with false discovery rate correction, principal component analysis (PCA), hierarchical clustering, and bootstrap stability analysis were performed.

**Results:**

The first 2 principal components explained 77.7% of the total variance. The first principal component (PC1) was mainly associated with relaxation-related parameters and diffusion kurtosis metrics, whereas the second principal component (PC2) was characterized by opposing contributions from AK and FA. Quantitative susceptibility mapping showed weak correlations with other parameters (*r* = −0.37 to −0.08), suggesting relatively independent susceptibility-related information. The PCA structure was preserved after excluding R_1_·R_2_*, and bootstrap analysis supported loading stability, with mean loading correlations of 0.94 for PC1 and 0.90 for PC2. WM tracts formed 4 major cluster-like groups and showed tract-specific multimodal fingerprints.

**Conclusion:**

Multimodal quantitative MRI provides a concise tract-level MRI feature representation of WM by integrating relaxation, susceptibility, and diffusion information. This exploratory framework may support future studies investigating subtle WM alterations, although validation in pathological cohorts is required.

## Introduction

White matter (WM) plays a crucial role in brain connectivity and information transfer, and its microstructural alterations are associated with a variety of neurological disorders. However, subtle abnormalities in WM are not always detectable on conventional MRI, and pathological changes may be present even in normal-appearing white matter (NAWM).^1^ A systematic characterization of normal WM is therefore an essential prerequisite for developing sensitive imaging biomarkers for disease. Advanced quantitative MRI techniques offer a means to characterize WM tissue properties beyond conventional image contrast.

Several quantitative MRI approaches have been proposed to evaluate WM tissue properties. Diffusion-based methods, such as diffusion tensor imaging (DTI)^2^ and diffusion kurtosis imaging (DKI),^3^ provide information on microstructural organization by measuring water diffusion characteristics. Parameters including fractional anisotropy (FA), mean kurtosis (MK), axial kurtosis (AK), and radial kurtosis (RK) have been widely used to assess fiber orientation, tissue complexity, and microstructural heterogeneity.^4^ In addition to diffusion imaging, relaxometry-based techniques allow quantitative estimation of relaxation parameters that reflect tissue composition. Quantitative parameter mapping (QPM), also referred to as synthetic MRI-based mapping, enables simultaneous estimation of multiple parameters including T_1_, T_2_*, proton density, and B_1_ using optimized multi-echo gradient-echo acquisitions.^5^ These relaxation parameters are known to be influenced by myelin content, macromolecular composition, and magnetic susceptibility effects.^6^^–^^8^

Another important quantitative MRI approach is quantitative susceptibility mapping (QSM), which provides information about tissue magnetic susceptibility and is sensitive to the presence of paramagnetic and diamagnetic substances such as iron and myelin. Previous studies have demonstrated that susceptibility contrast can reflect variations in myelin and iron distribution in WM structures.^7^^,^^9^ Furthermore, recent studies have proposed myelin-related imaging metrics derived from combinations of relaxation parameters, such as the product of relaxation rates (R_1_· R_2_*), which has been suggested as a surrogate marker of myelin content.^10^^–^^12^

Although each of these quantitative MRI techniques provides valuable information, they capture different aspects of tissue microstructure.^6^^–^^8^ Diffusion metrics primarily reflect microstructural organization and fiber orientation,^13^ whereas relaxation parameters are influenced by tissue composition and macromolecular content.^6^^,^^8^ Susceptibility measures are sensitive to magnetic properties related to myelin and iron.^7^^,^^14^ Therefore, a comprehensive understanding of WM microstructure likely requires the integration of multiple quantitative MRI parameters. However, the relationships among relaxation-based, susceptibility-based, and diffusion-based parameters across different WM structures remain insufficiently characterized.

It remains unclear how many quantitative features are required to adequately describe the structural characteristics of WM. Understanding the redundancy and complementarity among these parameters may help optimize the selection of imaging biomarkers for the evaluation of WM integrity. Furthermore, systematic comparison of these quantitative parameters across anatomically defined WM tracts may reveal characteristic patterns associated with specific fiber systems.

The purpose of this study was to investigate how relaxation-, susceptibility-, and diffusion-derived MRI parameters are organized across healthy WM tracts using a tract-wise multimodal framework. Quantitative values were extracted from atlas-defined WM tracts, and multivariate analyses were performed to evaluate redundancy, complementarity, and tract-specific MRI feature patterns among the parameters. This exploratory study aimed to determine whether multimodal quantitative MRI can provide a concise tract-level representation of WM feature organization.

## Materials and Methods

### Participants

Twenty-two healthy volunteers (15 males and 7 females; mean age, 22.8 ± 5.1 years; age range, 18–43 years) participated in this study. None of the participants had a history of neurological or psychiatric disorders. The study protocol was approved by the ethics committee of Tokushima University Hospital (#2275), and written informed consent was obtained from all participants prior to the MRI examination.

### MRI acquisition

MRI examinations were performed using a 3T MRI system (FUJIFILM, Tokyo, Japan) equipped with a 32-channel head coil. QPM datasets were acquired using a 3D RF spoiled steady-state gradient echo sequence. Imaging parameters were as follows: TRs were 10 and 40 ms. The TE settings were not identical across all acquisitions; the shortest TE was 4.6 ms, and the multi-echo acquisitions included TEs up to 36.8 ms with an echo spacing of 4.6 ms; flip angles, 10, 20, 32.1, and 41.3 degrees; RF phases, 160, 162, 172, and 173 degrees. The voxel size for QPM was 0.9 × 0.9 × 1.0 mm^3^.

Diffusion MRI datasets were acquired using a single-shot echo-planar imaging (EPI) sequence with the following parameters: TE, 110 ms; TR, 6500 ms; flip angle = 90 degrees, and voxel size = 1.9 × 1.9 × 2.0 mm^3^. Diffusion-weighted images (DWIs) were obtained with multiple *b*-values: 0, 800, and 2500 s/mm^2^ in 21 diffusion gradient directions. The diffusion encoding scheme was selected based on prior work optimizing DKI acquisition parameters, including *b*-value selection, number of diffusion directions, and scan-time reduction for reliable DKI metric estimation.^15^

The acquisition time was approximately 10 minutes for QPM and 9 minutes for DKI, resulting in a total scan time of approximately 19 minutes.

### Quantitative map reconstruction and preprocessing

The overall image-processing and analysis workflow is summarized in Fig. 1. Quantitative maps of R_1_ (1/T_1_), R_2_*(1/T_2_*), and R_1_· R_2_* were generated from the QPM dataset using a previously described QPM reconstruction framework.^5^^,^^10^^,^^11^ QSM was reconstructed from the phase images derived from the same dataset using phase unwrapping, background field removal, and dipole inversion.^16^

DKI maps were generated on the MRI system using vendor-provided processing software (FUJIFILM, Tokyo, Japan). DWIs acquired with *b*-values of 0, 800, and 2500 s/mm^2^ were used to estimate the diffusion and kurtosis tensors, from which FA, MK, AK, and RK maps were generated. FA was derived from the diffusion tensor estimated during this vendor-provided DKI processing. No additional offline diffusion preprocessing, such as denoising, Gibbs-ringing removal, EPI distortion correction, eddy-current correction, or bias-field correction, was performed before tract-wise analysis.

After map generation, diffusion-derived maps were visually inspected for gross artifacts and coregistered to the QPM-derived T_1_-weighted image. Prior to spatial normalization, a brain mask was generated from the QPM-derived T_1-_weighted image and applied to each quantitative parameter map to exclude non-brain tissues. All quantitative parameter maps were spatially normalized to the MNI standard space using FMRIB Software Library (FSL) software^17^^–^^19^ and resampled to 2.0 × 2.0 × 2.0 mm^3^ for Johns Hopkins University (JHU) atlas–based tract-wise analysis. Visual quality control was performed to confirm the adequacy of coregistration, spatial normalization, and atlas alignment.

### Spatial normalization and feature extraction

WM regions were defined using the JHU White Matter Atlas, which provides anatomically defined WM tracts based on probabilistic tractography. A total of 48 WM tracts were included in the present analysis.

For each subject and each WM tract, the mean values of the following quantitative parameters were extracted: R_1_, R_2_*, R_1_· R_2_*, QSM, FA, MK, AK, and RK. Thus, each WM tract was characterized by 8 quantitative MRI parameters reflecting relaxation-, susceptibility-, and diffusion-derived MRI features. For each tract and parameter, age-adjusted mean values were estimated across participants by including age as a covariate before constructing the group-level feature matrix. For tract-based multivariate analyses, values were then bilaterally averaged for each pair of homologous atlas-defined WM tracts, whereas unpaired midline tracts were retained individually. Accordingly, each observation used in the subsequent principal component analysis (PCA), clustering, similarity analysis, and radar-plot visualization corresponded to 1 WM tract represented by an 8-dimensional feature vector, after age adjustment and left-right averaging where applicable. To enable comparison across parameters with different physical units and dynamic ranges, all features were standardized prior to multivariate analyses. Standardization was performed for each parameter across WM tracts using z-score normalization:

(1) $$z=\frac{x-\mu }{\sigma },$$

where x represents the original parameter value, μ denotes the mean across observations, and σ represents the standard deviation. This normalization ensured that all parameters contributed equally to subsequent multivariate analyses.

### Statistical and multivariate analysis

To investigate the relationships among the quantitative MRI parameters, Pearson correlation analysis was performed using the regional mean values obtained from the bilaterally averaged WM tracts. This analysis was used to assess redundancy and complementarity among relaxation-based, susceptibility-based, and diffusion-based parameters across WM structures. Pearson correlation coefficients were calculated for all pairwise combinations of quantitative MRI parameters using bilaterally averaged tract-level values. To account for multiple comparisons across all pairwise correlations, the false discovery rate (FDR) was controlled using the Benjamini–Hochberg procedure, and FDR-adjusted *q*-values were computed. Correlation coefficients and their statistical significance after FDR correction were visualized using an upper-triangular correlation matrix.

For network visualization, correlations were thresholded based on effect size (|r| > 0.4) rather than statistical significance, in order to highlight structurally meaningful relationships among parameters. The corresponding *P*-values and FDR-adjusted *q*-values were additionally calculated to ensure statistical validity.

To evaluate the internal stability of the PCA loading pattern within the present dataset, bootstrap resampling was performed by resampling bilaterally averaged WM tracts with replacement. For each bootstrap sample, PCA was repeated using the standardized feature matrix. The loading vectors obtained from each bootstrap sample were compared with those from the original PCA solution using Pearson correlation coefficients after sign alignment. This analysis evaluated the stability of PCA loading patterns within the present tract-level dataset and was not intended to assess test–retest reproducibility, inter-scanner reproducibility, or external generalizability.

### PCA

PCA was performed across atlas-defined WM tracts to characterize tract-level variation in multimodal MRI features; therefore, regional variability among tracts was the primary source of variation evaluated in this analysis. PCA was applied to the matrix of bilaterally averaged WM tracts and 8 standardized quantitative parameters to identify dominant patterns within the multimodal MRI feature space. First, the covariance matrix of the standardized feature matrix Z was calculated as:

(2) $$\sum^{\text{​}}=\frac{1}{n-1}Z^{T}Z.$$

Eigenvalue decomposition was then performed:

(3) ∑v=λv,

where v represents eigenvectors corresponding to principal component directions and λ denotes eigenvalues representing the variance explained by each component. The resulting principal components provide orthogonal feature axes describing the dominant variance patterns in the multimodal MRI dataset. Loading plots were used to evaluate the contribution of each parameter to the principal components and to interpret the resulting MRI feature patterns.

### WM fingerprint representation

To visualize tract-specific multimodal fingerprints, the subject-level feature vectors were averaged across participants for each bilaterally averaged WM tract. The resulting group-level tract fingerprint was defined as:

(4) $$F_{WM}=\left(R_{1},R_{2}^{*},R_{1}\cdot R_{2}^{*},QSM,FA,MK,AK,RK\right).$$

These feature vectors describe the integrated MRI characteristics of each WM structure. Tract-wise mean fingerprints were calculated across subjects and visualized using radar plots to illustrate differences in parameter profiles among representative WM tracts. Four representative tracts (genu of the corpus callosum [GCC], posterior limb of the internal capsule [PLIC], anterior corona radiata [ACR], and superior corona radiata [SCR]) were selected to illustrate distinct multimodal feature profiles across anatomically different WM tracts.

### Hierarchical clustering analysis

To examine similarities among WM tracts based on their multimodal MRI characteristics, hierarchical clustering analysis was performed. The similarity between 2 WM tracts i and j was evaluated using Euclidean distance:

(5) $$d_{ij}=\sqrt{\overset{p}{\underset{k=1}{\sum }}{\left(x_{ik}-x_{jk}\right)}^{2}},$$

where p represents the number of parameters. Agglomerative clustering was performed using the Ward linkage method, which minimizes the variance within clusters. The resulting dendrogram provided a hierarchical representation of similarities among WM tracts. In addition to hierarchical clustering, a tract-by-tract similarity matrix was generated using Pearson correlation coefficients between the 8-dimensional multimodal feature vectors of the WM tracts. The resulting similarity values ranged from −1 to 1 and were visualized as a heatmap to assess the similarity structure among tracts. For descriptive interpretation, 4 major clusters were defined according to the visually identifiable block structure in the similarity matrix and the corresponding dendrogram. This 4-cluster partition was used as a descriptive aid for visualization and interpretation, and the main conclusions of the present study do not depend on the exact number of clusters.

Statistical analyses, including correlation analysis and principal component analysis, were performed using JMP software (SAS Institute, Cary, NC, USA). Additional multivariate analyses, data processing, and figure generation were performed using MATLAB (MathWorks, Natick, MA, USA).

## Results

### Overview of multimodal quantitative MRI features

Group-averaged multimodal quantitative MRI maps derived from QPM and diffusion MRI are shown in Fig. 2. Representative native-space brain-masked maps before spatial normalization are provided in Supplementary Fig. S1. Relaxation-based parameters (R_1_, R_2_*, and R_1_· R_2_*) exhibited distinct contrast patterns reflecting tissue composition, while QSM demonstrated susceptibility variations associated with underlying microstructural properties. Diffusion-based metrics (FA, MK, AK, and RK) highlighted differences in fiber organization and tissue complexity. The displayed parametric maps covered 8 quantitative features in total, integrating relaxation, susceptibility, and diffusion information within the same framework. Overall, each parameter provided complementary spatial information, indicating that WM microstructure cannot be fully characterized by a single modality.

### Principal component structure of WM features

PCA of bilaterally averaged WM tracts revealed that the multimodal MRI features could be represented by a small number of dominant components (Fig. 3). The first principal component (PC1) explained approximately 59.1% of the total variance, while the second component (PC2) accounted for approximately 18.6%, indicating that most of the variability across WM tracts was captured by the first 2 components. Together, PC1 and PC2 explained 77.7% of the total variance. The third principal component explained approximately 11.3%, and the cumulative explained variance reached approximately 88.9% by the first 3 components and 95.4% by the first 4 components. The loading plot showed that diffusion-related parameters (FA, MK, and RK) and relaxation-related parameters contributed differently to the principal components, suggesting that they reflect different aspects of WM microstructure. AK showed the largest positive loading along PC2, whereas FA was located on the negative side of PC2. By contrast, R_1_, R_2_*, R_1_· R_2_*, MK, and RK were positioned mainly on the positive side of PC1, indicating shared contributions to the dominant pattern of variance. QSM was positioned on the opposite side of PC1, suggesting a contribution distinct from the other parameters. These results suggest that multimodal MRI features contain both overlapping and complementary information across modalities.

### Inter-parameter relationships and redundancy

The relationships among quantitative MRI parameters were further evaluated using correlation analysis (Fig. 4). As shown in the correlation matrix (Fig. 4a), strong positive correlations were observed among relaxation-based parameters, indicating substantial redundancy within this modality. All pairwise correlation coefficients, corresponding *P*-values, and FDR-adjusted *q*-values are provided in Supplementary Table S1. R_1_ correlated positively with R_2_* (*r* = 0.58) and R_1_· R_2_* (*r* = 0.83), while the strongest association within this group was observed between R_2_* and R1· R_2_* (r = 0.85). Diffusion-related parameters also showed strong inter-correlations, most notably between MK and RK (r = 0.92) and between FA and RK (*r* = 0.85), with a moderate positive correlation between FA and MK (*r* = 0.61). By contrast, AK showed a weaker association with RK (*r* = 0.26), suggesting partial dissociation within diffusion-derived measures. In contrast to these clustered relationships, QSM exhibited only weak correlations with the other parameters, with coefficients ranging from −0.37 to −0.08. Negative correlations were observed between QSM and relaxation-related parameters, including R_1_ (*r* = −0.37), R_2_* (*r* = −0.28), and R_1_· R_2_* (*r* = −0.37), as well as with diffusion-related parameters such as FA (*r* = −0.17), MK (*r* = −0.26), AK (*r* = −0.08), and RK (*r* = −0.25). The network representation (Fig. 4b), constructed using an effect-size threshold (|r| > 0.4) to highlight moderate-to-strong associations, demonstrated a similar structure, revealing partially separable relaxation- and diffusion-related modules while highlighting the relative independence of QSM. Several of the strongest correlations remained significant after FDR correction.

### Internal stability of the low-dimensional feature structure

To evaluate whether the identified PCA structure was driven primarily by the mathematically coupled R_1_· R_2_* parameter, PCA was repeated after excluding R_1_· R_2_*. The overall loading pattern was largely preserved, with QSM remaining relatively isolated and diffusion-related metrics maintaining a similar distribution in principal component (PC) space (Fig. 5a). Bootstrap resampling demonstrated high internal stability of the PCA loading pattern within the present dataset, particularly for PC1, with mean loading correlations of 0.94 for PC1 and 0.90 for PC2 relative to the original PCA solution (Fig. 5b).

### Spatial heterogeneity across white matter tracts

The distribution of multimodal MRI features across bilaterally averaged WM tracts is summarized in the z-score heatmap (Fig. 6). Distinct tract-specific patterns were observed, indicating heterogeneity in WM microstructure. The standardized values spanned approximately −2.5 to +2.5, demonstrating variation among anatomically defined tracts. Projection-related tracts such as the PLIC tended to show relatively high FA/RK, whereas limbic or commissural tracts exhibited different combinations of relaxation and susceptibility features. Hierarchical clustering further revealed structured relationships among WM tracts with similar multimodal feature profiles.

### Clustering of WM structures based on multimodal features

To further investigate structural similarity among bilaterally averaged WM tracts, a similarity matrix was constructed based on multimodal features (Fig. 7). The similarity values ranged from −1 to +1, with strong positive similarity within clusters and lower or negative similarity between clusters. The similarity matrix showed 4 major cluster-like blocks, indicating structured organization in multimodal WM feature space. These groups were interpreted post hoc as loosely overlapping with known anatomical systems, although the clustering was not strictly anatomical because it was driven by multimodal feature similarity rather than anatomical connectivity alone. Tracts within the same cluster exhibited high similarity, whereas inter-cluster similarity was markedly lower.

### Regional multi-parametric fingerprints of representative tracts

Radar plots of representative bilaterally averaged WM tracts demonstrated distinct multimodal fingerprints (Fig. 8). For example, the PLIC showed high diffusion anisotropy and kurtosis values, whereas the GCC exhibited a more balanced profile across parameters. Representative tracts such as the ACR and SCR displayed characteristic patterns distinct from GCC and PLIC. The radial scale of the radar plots ranged approximately from −1.2 to +1.2, and the observed deviations across this range indicate that each tract possesses a unique combination of relaxation, susceptibility, and diffusion properties. These results highlight that each WM tract possesses a distinct quantitative MRI fingerprint, enabling tract-specific characterization.

## Discussion

### Principal findings

In this study, we performed an integrative analysis of relaxation, susceptibility, and diffusion MRI parameters to characterize WM structure in a unified multimodal framework. The main findings can be summarized as follows. First, the 8 quantitative MRI parameters could be represented in a low-dimensional space, with the first 2 principal components explaining approximately 78% of the total variance. Second, substantial redundancy was observed among several parameters, particularly within relaxation- and diffusion-based metrics, whereas QSM exhibited relative independence from the other parameters. Third, WM tracts formed structured clusters and exhibited tract-specific multimodal fingerprints, indicating that integrated MRI features captured organized tract-level feature patterns. Importantly, these findings collectively support a tract-based multimodal framework in which complementary MRI contrasts are integrated into a compact tract-level representation of WM MRI feature organization.

### Low-dimensional organization of multimodal MRI feature patterns

A central finding of this study is that multimodal MRI features of WM can be effectively represented in a low-dimensional feature space (Fig. 3). Despite the use of 8 quantitative parameters, the majority of variance across WM tracts was explained by only 2 principal components. This result suggests that the apparent complexity of multimodal MRI measurements may be summarized by a smaller number of MRI feature patterns rather than 8 fully independent dimensions. This low-dimensional organization was further supported by the additional analyses. The overall PCA loading pattern remained largely preserved even after exclusion of the mathematically coupled parameter R_1_· R_2_* (Fig. 5a). This additional analysis was performed because R_1_· R_2_* is mathematically derived from R_1_ and R_2_* and could therefore influence the apparent low-dimensional structure. Bootstrap resampling further suggested internal stability of the PCA loading pattern within the present tract-level dataset (Fig. 5b). However, these analyses do not demonstrate test–retest reproducibility, inter-scanner reproducibility, or external generalizability. Therefore, the present findings should be interpreted as exploratory MRI feature patterns observed in this dataset, rather than as directly validated biological axes.

PC1 (Fig. 3b), which was primarily associated with relaxation-related parameters (R_1_, R_2_*, and R_1_· R_2_*) together with diffusion kurtosis metrics such as MK and RK, may be related to tissue compositional properties, particularly those associated with myelin and macromolecular density.^6^^,^^8^^,^^10^ These parameters are known to be influenced by myelin content, water compartmentalization, and interactions between water and macromolecules.^6^^,^^8^ Their convergence along PC1 therefore suggests a relaxation- and kurtosis-dominant MRI feature pattern that may be associated with compositional variation in WM. However, because the present study did not include histological validation or biophysical model fitting, this interpretation should be regarded as hypothesis-generating rather than as direct evidence of a specific biological process.

In contrast, PC2 (Fig. 3b), characterized by opposing contributions from parameters such as AK and FA, may be related to diffusion-based feature variation associated with fiber geometry, orientation dispersion, and structural anisotropy. This pattern is consistent with known effects of fiber architecture, in which increased crossing fibers or orientation dispersion typically reduce FA while altering kurtosis behavior.^13^ Accordingly, PC2 may capture a diffusion organization–related MRI feature pattern that is not directly explained by bulk tissue compositional properties alone. Nevertheless, PC2 should also be interpreted cautiously because diffusion-derived metrics can be affected by multiple biological and technical factors, including fiber orientation, crossing fibers, partial-volume effects, and acquisition-related biases.

Importantly, QSM exhibited weak correlations with both PC1- and PC2-associated parameters, indicating that magnetic susceptibility provides an additional and relatively independent source of contrast.^7^^,^^14^ Unlike relaxation and diffusion metrics, which are mainly sensitive to water dynamics and microstructural organization, QSM is sensitive to the underlying magnetic susceptibility sources, including diamagnetic components such as myelin and paramagnetic substances such as iron.^7^ This interpretation was further supported by the correlation and network analyses (Fig. 4), in which QSM showed only weak pairwise associations with the other parameters and limited connectivity relative to the relaxation- and diffusion-related modules. Rather than simply behaving as an isolated parameter, QSM may therefore represent a complementary susceptibility-sensitive MRI feature that is not reducible to relaxation- or diffusion-based contrasts alone. Recent advances in susceptibility imaging, including χ-separation techniques, further suggest that this susceptibility-sensitive information can itself be decomposed into biologically distinct components.^20^^,^^21^ Taken together, these findings suggest a tract-wise multimodal MRI framework (Figs. 3 and 4) in which WM feature organization can be explored from at least 3 complementary perspectives: relaxation/kurtosis–dominant, diffusion organization–related, and susceptibility–sensitive feature patterns.

Such a multidimensional framework may be particularly valuable in future studies aimed at detecting subtle abnormalities in NAWM, where conventional MRI contrast often remains insensitive to early or diffuse pathological changes.^22^ By integrating complementary MRI feature information related to tissue composition, fiber organization, and magnetic susceptibility, multimodal feature fingerprints (Fig. 8) may provide a more sensitive representation of WM integrity than single-parameter approaches.

### Structural organization and multimodal clustering of WM

The clustering and similarity analyses (Fig. 7) demonstrated that WM tracts were not randomly distributed in the multimodal feature space, but instead formed structured groups. These cluster-like patterns suggest that multimodal MRI features capture organized variation across WM tracts. Although the observed groupings were interpreted post hoc as broadly corresponding to known anatomical systems, they did not strictly follow classical anatomical classifications, likely because the clustering was driven by similarities in multimodal microstructural features rather than anatomical connectivity alone. Importantly, this clustering emerged without explicit anatomical constraints, indicating that the multimodal feature space preserves meaningful relationships among WM tracts. This observation supports the utility of the proposed feature extraction framework and suggests that multimodal MRI may provide data-driven representations of WM organization, consistent with previous multivariate approaches.^23^^,^^24^ Furthermore, the regional fingerprints (Fig. 8) revealed that each tract was characterized by a distinct combination of quantitative MRI parameters. These tract-specific profiles provide a compact representation of WM properties and may serve as a basis for future comparative studies of WM abnormalities.

### Implications for multimodal MRI and biomarker design

The present findings have important implications for the design of quantitative MRI protocols and the development of imaging biomarkers. First, the low-dimensional structure identified in this study (Fig. 3) suggests that a reduced set of parameters may be sufficient to capture the major multimodal MRI feature patterns of WM. This has potential benefits for reducing acquisition time and simplifying analysis pipelines. Second, the identification of redundant and complementary parameters (Fig. 4) provides a framework for rational feature selection and protocol optimization. Highly correlated parameters within the same modality may be interchangeable, whereas parameters that retain relatively independent information, such as QSM (Fig. 4), may be prioritized in multimodal imaging protocols.^14^ However, the present study was not designed to define an optimal reduced parameter set, and future studies should formally evaluate whether reduced parameter combinations can preserve tract-level characterization or disease sensitivity. The total acquisition time of the present protocol also suggests potential feasibility for future clinical implementation. Third, the concept of multimodal fingerprints (Fig. 8) offers a novel approach to characterizing WM tracts. Rather than relying on single parameters, integrated feature profiles may provide more comprehensive MRI feature information. Indeed, the multidimensional framework proposed in this study may be particularly valuable for detecting subtle abnormalities in NAWM. In these regions, conventional MRI contrast often remains insensitive to early or diffuse pathological changes.^22^ By providing more comprehensive MRI feature profiles through tract-specific fingerprints, this approach holds significant potential for differentiating various pathological conditions where single-parameter measurements may overlap or remain limited. This framework may be particularly useful in future studies of multiple sclerosis, in which abnormalities in NAWM may precede overt lesion formation. Multimodal fingerprints may help detect such subtle alterations more comprehensively than single-parameter approaches. The acquisition time of the present protocol may be compatible with future clinical or research-oriented MRI examinations.

### Limitations and future directions

Several limitations should be acknowledged. First, the study population was relatively young. Although age was included as a covariate when estimating tract-level mean values, this adjustment cannot fully remove nonlinear age-related effects on myelination, iron accumulation, or diffusion properties. Therefore, the identified feature structure should be interpreted as an exploratory pattern observed in young healthy adults rather than as a normative model across the lifespan. Future studies including a broader age range are needed to determine how aging influences the multimodal feature organization of WM. Second, this study was conducted in a relatively small cohort of healthy subjects and was intended as a proof-of-concept investigation rather than a normative population study; therefore, the findings may not directly generalize to pathological conditions. In demyelinating and other pathological conditions, multimodal MRI features may be affected by demyelination, axonal injury, inflammation, edema, gliosis, and iron accumulation. Therefore, the present feature patterns should not be directly extrapolated to disease cohorts without further validation. Future studies are needed to evaluate whether the identified feature structures are preserved or altered in disease. Test–retest validation was not performed; therefore, the reproducibility of the identified tract-level feature organization remains to be established. Third, the analysis was based on atlas-defined WM tracts, which may not fully capture subject-specific anatomical variability. In addition, PCA and clustering results may be influenced by atlas definition, tract size, partial-volume effects, and regional registration accuracy. Voxel-wise or subject-specific analyses may provide additional insights into local microstructural heterogeneity. Fourth, although the multimodal MRI parameters are sensitive to underlying tissue properties, their biological interpretation remains indirect. QSM was treated as a scalar measure in this study, although WM susceptibility can exhibit orientation dependence. Thus, QSM variability may partly reflect fiber orientation relative to B_0_, in addition to myelin- or iron-related susceptibility sources. Although the additional analyses suggested internal stability of the PCA loading pattern (Fig. 5), they do not eliminate the possibility of systematic diffusion-encoding bias. In particular, all diffusion-derived metrics used in this study, including FA, MK, AK, and RK, ultimately originate from diffusion-weighted imaging signals. Therefore, systematic errors at the DWI acquisition level, such as gradient nonlinearity, spatial variability of the effective *b*-matrix, and diffusion-encoding inaccuracies, may propagate into DTI- and DKI-derived metrics.^25^^–^^28^ Such systematic effects are distinct from random noise and may not be fully addressed by standard preprocessing alone.^27^^,^^28^ Accordingly, the low-dimensional structure identified in this study should be interpreted as reflecting both biological variation and potential acquisition-related sensitivities. Future studies using physically corrected diffusion metrics, calibration-based approaches, and test–retest data will be important to further validate the proposed framework. In addition, further validation using histological or biophysical models would be valuable to clarify the relationship between MRI-derived features and tissue microstructure. Finally, the present framework focused on a limited set of quantitative MRI parameters. While the use of standardized feature representations may support cross-dataset comparability, further studies are needed to determine whether the proposed multimodal fingerprint can be consistently reproduced across different scanners and institutions. The inclusion of additional modalities, such as myelin water imaging^29^ or advanced diffusion models,^30^ may further enhance the characterization of WM structure.

## Conclusion

In conclusion, multimodal quantitative MRI provided a compact tract-level representation of WM MRI feature organization in young healthy adults. The proposed exploratory framework identified low-dimensional feature patterns, redundancy and complementarity among parameters, and tract-specific multimodal profiles. These findings support the feasibility of tract-wise multimodal MRI feature analysis, while further validation in larger, age-diverse, pathological, and multi-scanner cohorts is required.

## Supplementary Materials

Supplementary figures are available online.

**Figure:**
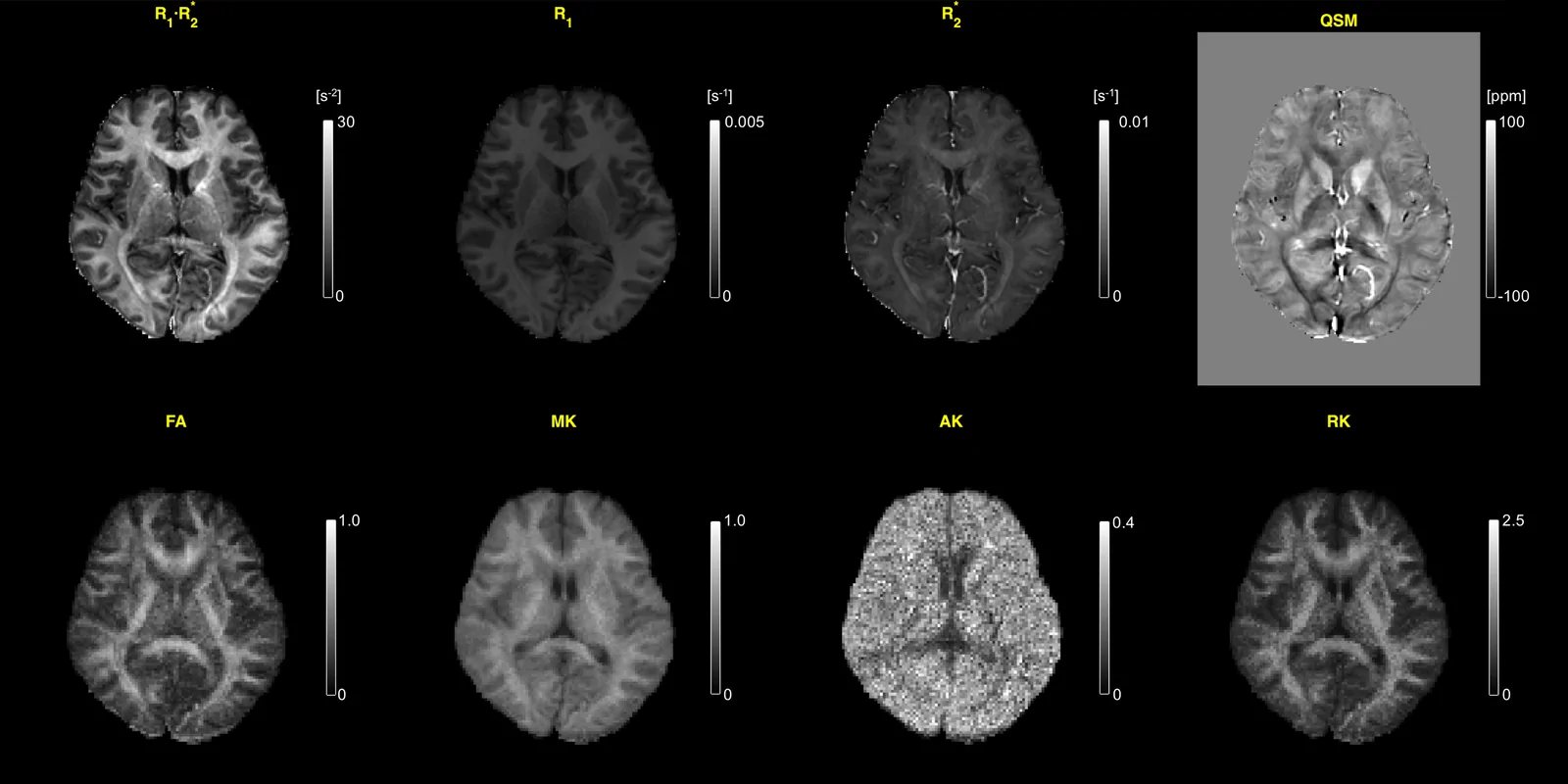


**Supplementary Table S1:**
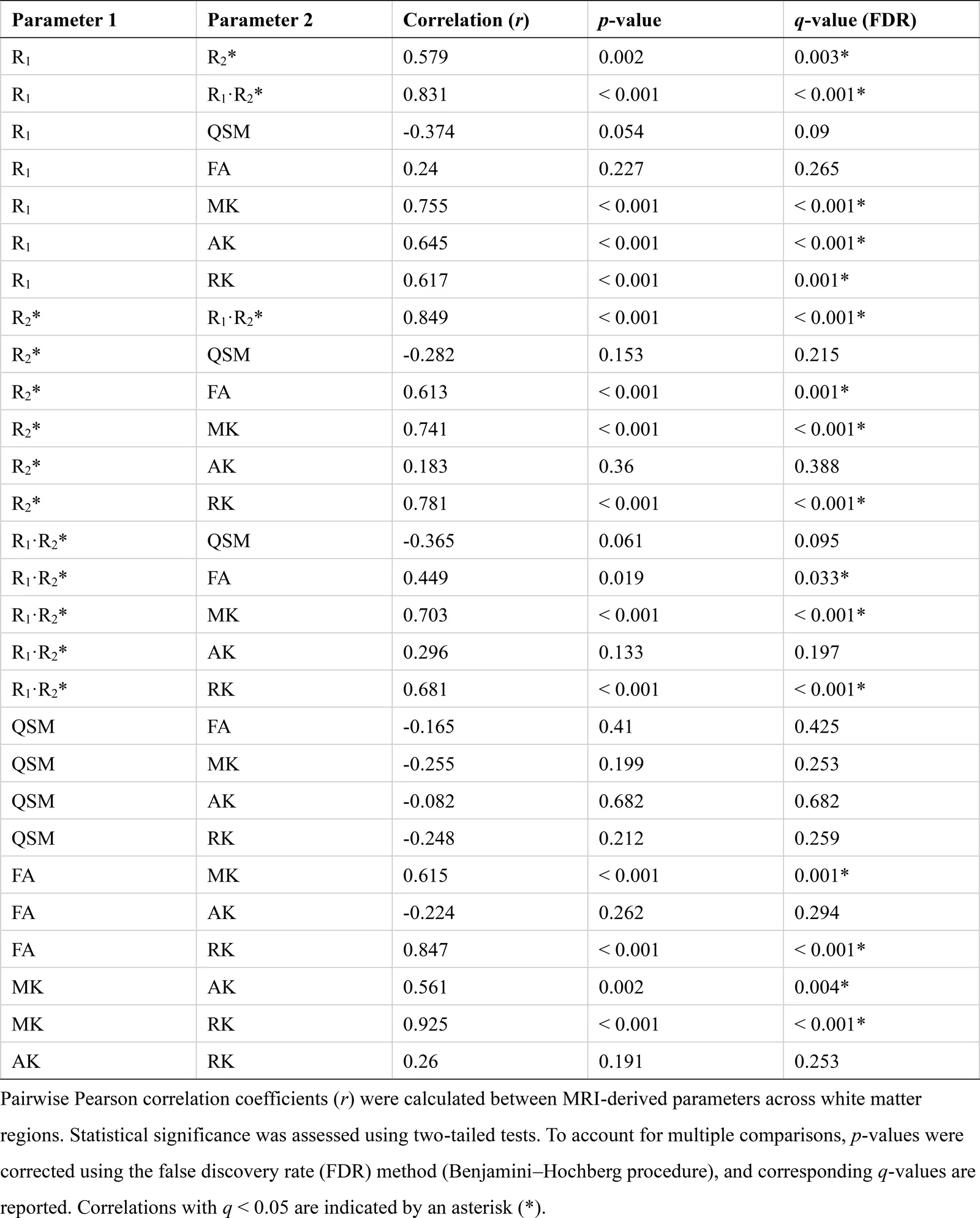
Pairwise correlations among MRI parameters

## Figures and Tables

**Fig. 1:**
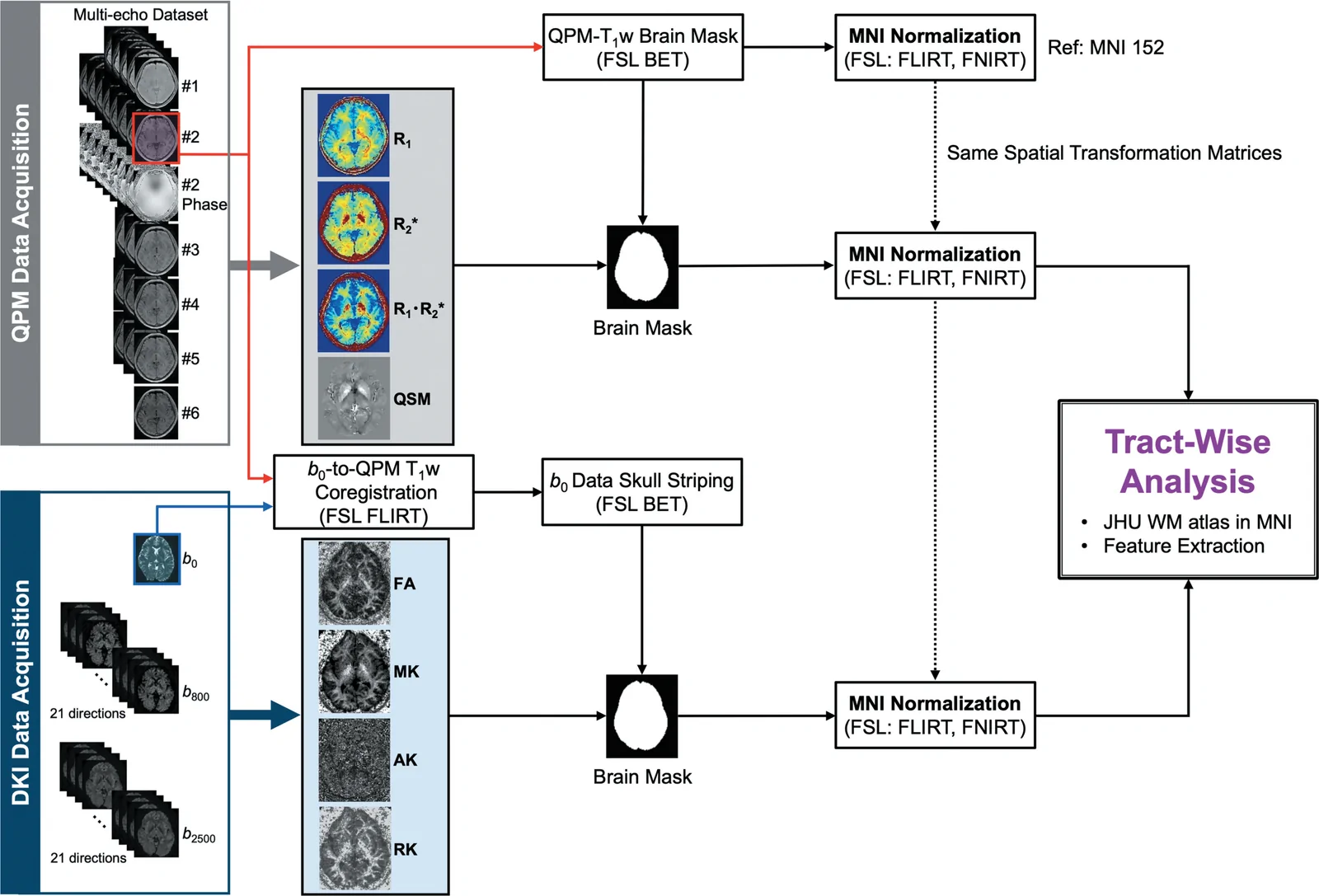
Overview of the multimodal MRI analysis pipeline. QPM data were used to derive relaxation parameters (R_1_, R_2_*, and R_1_· R_2_*) and QSM, while diffusion MRI data were used to compute diffusion metrics (FA, MK, AK, and RK). Brain masks were generated from QPM-derived T_1_-weighted images and applied to all parameter maps. Diffusion data were coregistered to the QPM-derived T_1_-weighted image, and all maps were spatially normalized to the MNI152 template using FSL (FLIRT, FNIRT). WM tracts were defined using the JHU WM atlas in MNI space, and tract-wise quantitative values were extracted for subsequent multivariate analyses. AK, axial kurtosis; DKI, diffusion kurtosis imaging; FA, fractional anisotropy; FLIRT, FMRIB’s Linear Image Registration Tool; FNIRT, FMRIB’s Nonlinear Image Registration Tool; FSL, FMRIB Software Library; JHU, Johns Hopkins University; MK, mean kurtosis; MNI, Montreal Neurological Institute; QPM, quantitative parameter mapping; QSM, quantitative susceptibility maps; RK, radial kurtosis; WM, white matter.

**Fig. 2:**
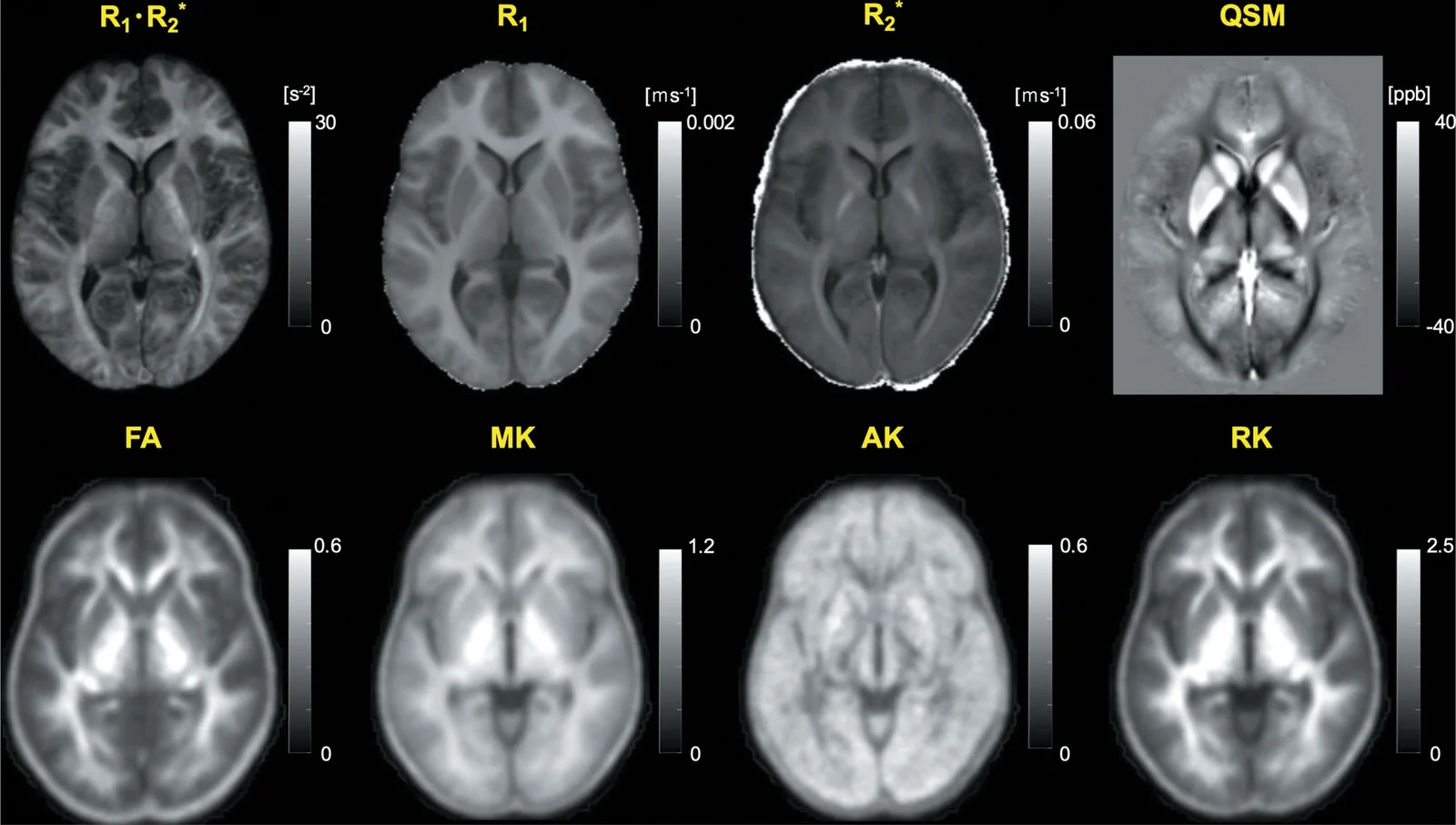
Group-averaged multimodal quantitative MRI maps in MNI space. Group-averaged relaxation-based maps (R_1_, R_2_*, and R_1_· R_2_*), susceptibility map (QSM), and diffusion-derived maps (FA, MK, AK, and RK) are shown after spatial normalization to MNI space. All maps are shown in the MNI space after preprocessing. These maps are presented in the same spatial framework used for subsequent JHU atlas–based tract-wise feature extraction and multivariate analyses. Each parameter provides complementary MRI feature information related to relaxation, susceptibility, and diffusion properties. AK, axial kurtosis; FA, fractional anisotropy; JHU, Johns Hopkins University; MK, mean kurtosis; MNU, Montreal Neurological Institute; QSM, quantitative susceptibility maps; RK, radial kurtosis.

**Fig. 3:**
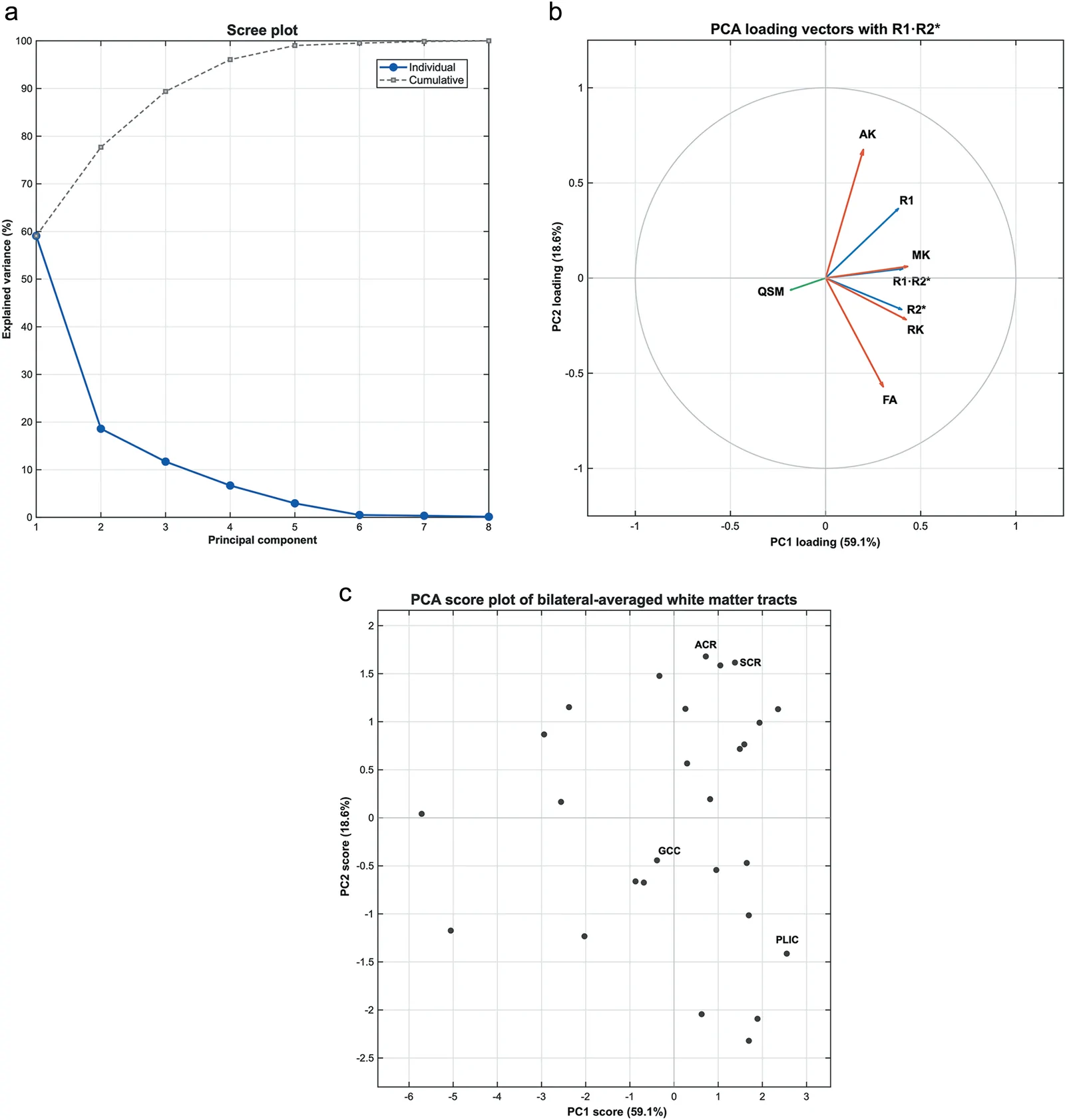
PCA of multimodal white matter features. (**a**) Scree plot illustrating the explained variance of each component and cumulative variance. (**b**) Loading plot showing the contribution of each quantitative MRI parameter to the principal components. (**c**) PCA score plot of bilaterally averaged white matter tracts in the space defined by the first 2 principal components (PC1 and PC2). ACR, anterior corona radiata; AK, axial kurtosis; FA, fractional anisotropy; GCC, genu of the corpus callosum; MK, mean kurtosis; PCA, principal component analysis, PC1, the first principal component; PC2, the second principal component; PLIC, posterior limb of the internal capsule; QSM, quantitative susceptibility maps; RK, radial kurtosis; SCR, superior corona radiata.

**Fig. 4:**
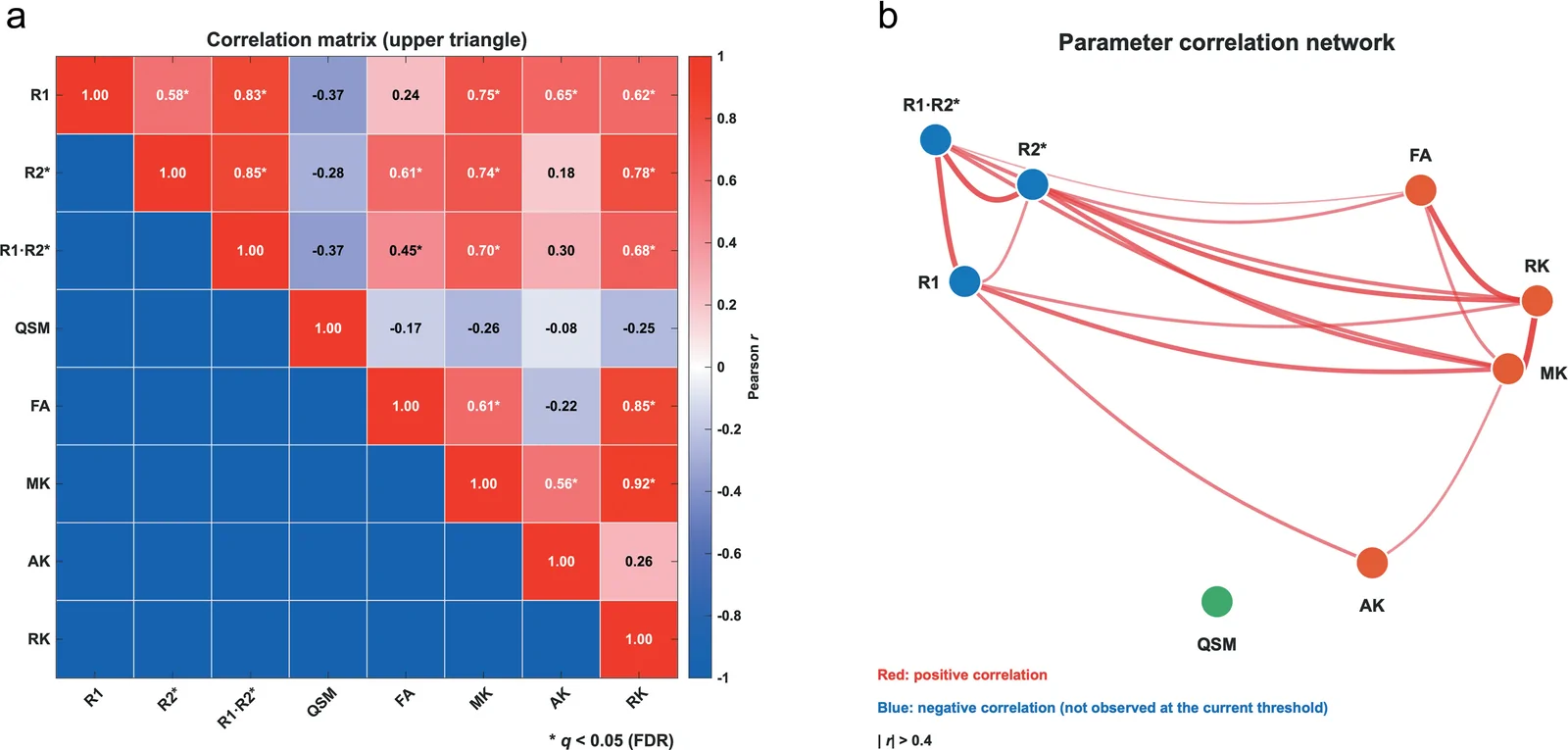
Multimodal relationships among MRI parameters. (**a**) Upper-triangular correlation matrix of the MRI parameters. Pearson correlation coefficients are shown, and statistically significant associations after FDR correction are marked with an asterisk. (**b**) Parameter correlation network based on bilaterally averaged white matter tracts. Edges represent correlations exceeding an effect-size threshold (|*r*| > 0.4). In the present data, only positive correlations met this threshold; therefore, all displayed edges are positive, and edge thickness reflects correlation magnitude. All pairwise correlation coefficients, corresponding *P*-values, and FDR-adjusted q-values are provided in Supplementary Table S1 for completeness. AK, axial kurtosis; FA, fractional anisotropy; FDR, false discovery rate; MK, mean kurtosis; QSM, quantitative susceptibility maps; RK, radial kurtosis.

**Fig. 5:**
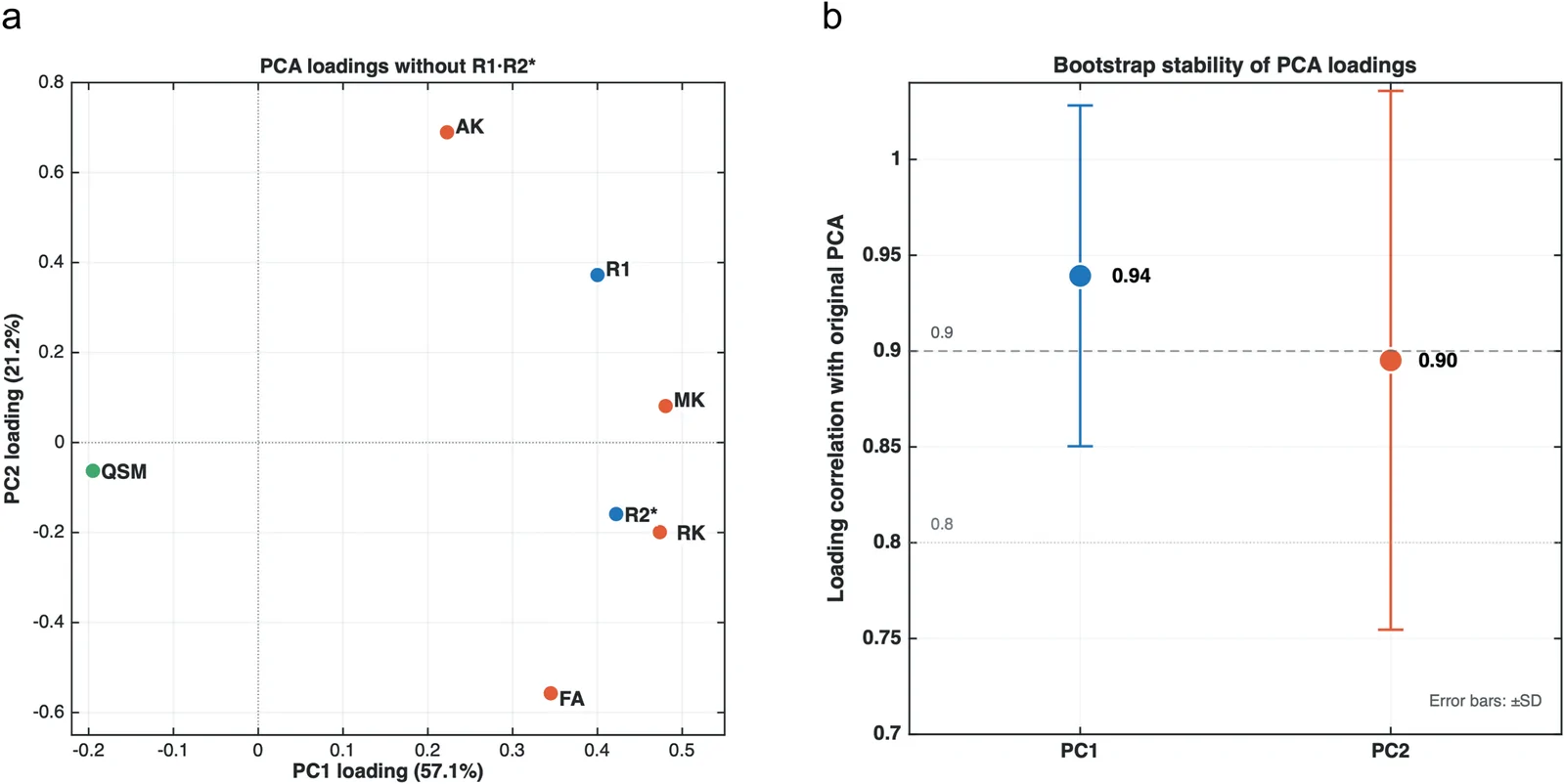
Internal stability analysis of the low-dimensional multimodal feature structure. (**a**) PCA loading plot after exclusion of the mathematically coupled parameter R_1_· R_2_*. The overall distribution of multimodal MRI parameters remained largely preserved, with QSM remaining relatively separated from the relaxation- and diffusion-related parameters. (**b**) Bootstrap stability analysis of PCA loadings. Points indicate mean loading correlations with the original PCA solution, and error bars indicate standard deviations across bootstrap resampling. PC1 showed particularly high stability, whereas PC2 showed greater variability but maintained a high mean loading correlation. AK, axial kurtosis; FA, fractional anisotropy; MK, mean kurtosis; PCA, principal component analysis; PC1, the first principal component; PC2, the second principal component; QSM, quantitative susceptibility mapping; RK, radial kurtosis.

**Fig. 6:**
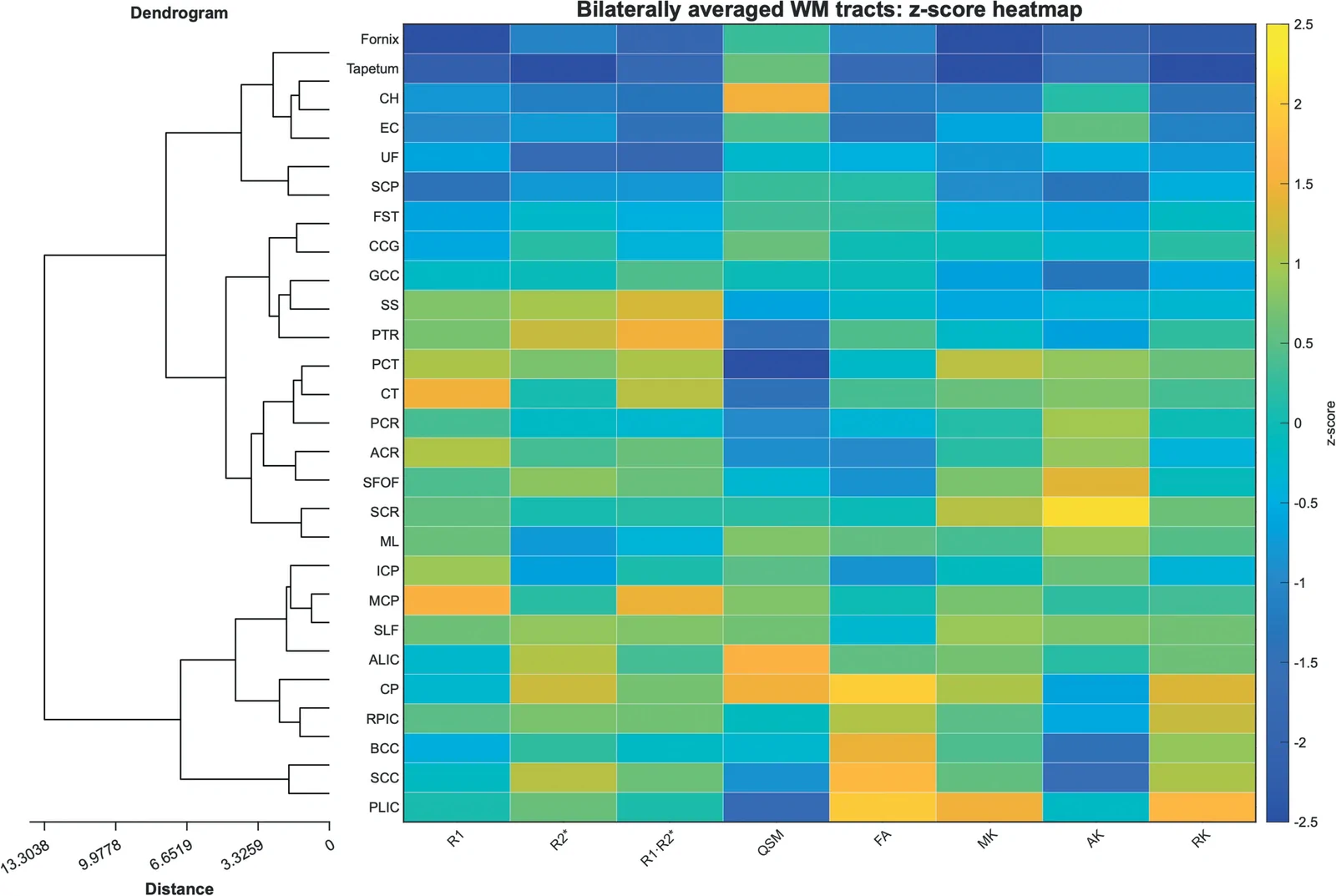
Regional distribution of multimodal MRI features across WM tracts. Z-score heatmap of 8 quantitative MRI parameters across bilaterally averaged WM tracts derived from the JHU atlas. Values were standardized across tracts. The accompanying dendrogram shows hierarchical clustering based on Euclidean distance and Ward linkage. Distinct regional patterns indicate structured variation in multimodal WM features. ACR, anterior corona radiata; AK, axial kurtosis; ALIC, anterior limb of internal capsule; BCC, body of corpus callosum; CCG, cingulate gyrus of corpus callosum; CH, cerebral hemisphere; CP, cerebral peduncle; EC, external capsule; FA, fractional anisotropy; FST, fronto-striatal tract; GCC, genu of the corpus callosum; ICP, inferior cerebellar peduncle; JHU, Johns Hopkins University, MK, mean kurtosis; ML, medial lemniscus; MCP, middle cerebellar peduncle; PCR, posterior corona radiat; PCT, pontine crossing tract; PLIC, posterior limb of the internal capsule; PTR, posterior thalamic radiation; QSM, quantitative susceptibility maps; RK, radial kurtosis, RPIC, retrolenticular part of internal capsule; SCC, splenium of corpus callosum; SCP, superior cerebellar peduncle; SCR, superior corona radiata; SFOF, superior fronto-occipital fasciculus; SLF, superior longitudinal fasciculus; SS; sagittal stratum; UF, uncinate fasciculus; WM, white matter.

**Fig. 7:**
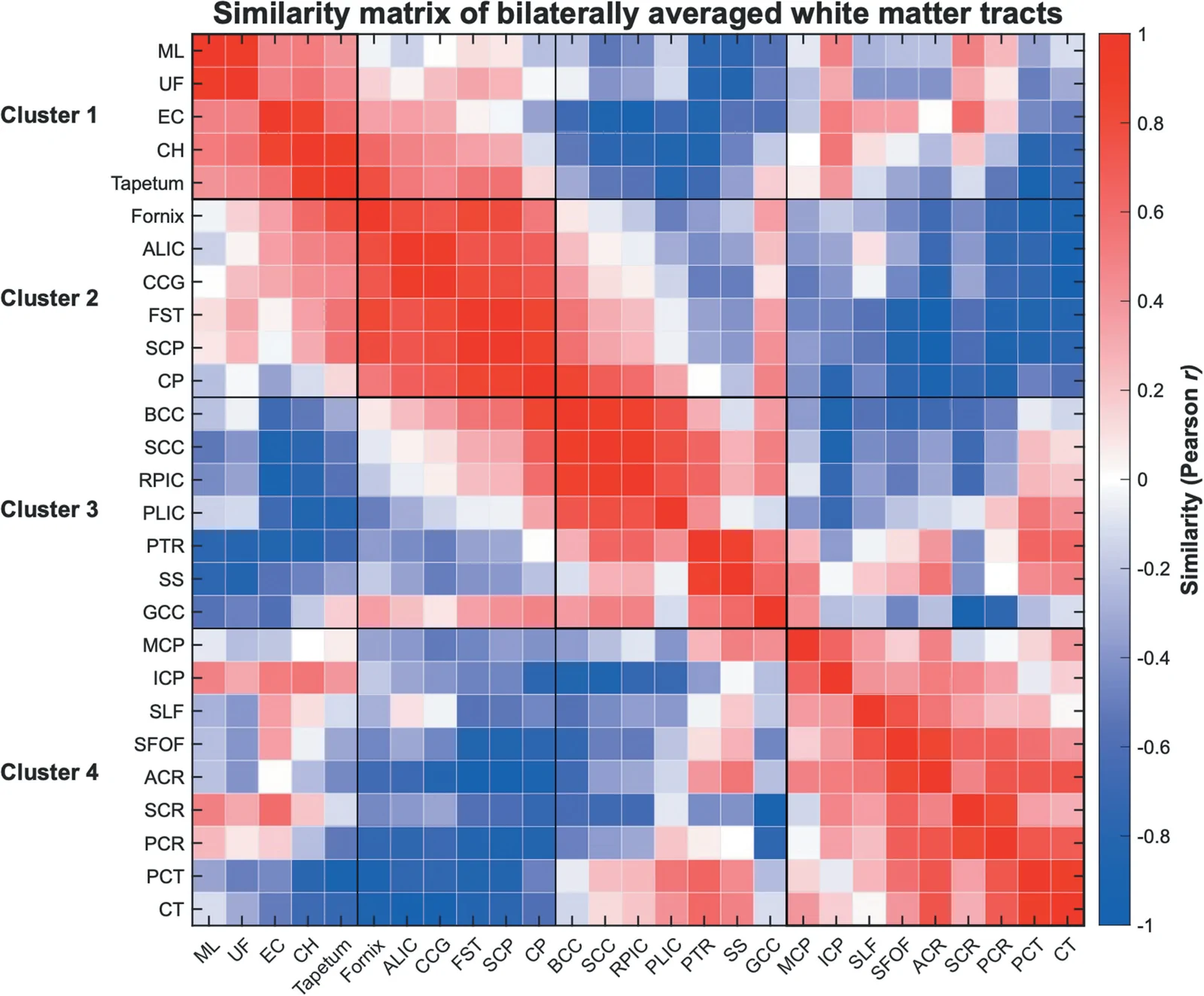
Similarity matrix of bilaterally averaged white matter tracts. Tract-by-tract similarity matrix based on Pearson correlation coefficients between bilaterally averaged multimodal feature vectors of white matter tracts. Values range from −1 to 1, with higher values indicating greater similarity. Four major cluster-like blocks are observed, indicating structured organization in multimodal white matter feature space. These groups were interpreted descriptively and only broadly related to known anatomical systems, although the clustering was not strictly anatomical because it was driven by multimodal feature similarity rather than anatomical connectivity alone. ACR, anterior corona radiata; ALIC, anterior limb of internal capsule; BCC, body of corpus callosum; CCG, cingulate gyrus of corpus callosum; CH, cerebral hemisphere; CP, cerebral peduncle; CT, corticospinal tract; EC, external capsule; FST, fronto-striatal tract; GCC, genu of corpus callosum; ICP, inferior cerebellar peduncle; MCP, middle cerebellar peduncle; ML, medial lemniscus; PCT, pontine crossing tract; PLIC, posterior limb of the internal capsule; PCR, posterior corona radiata; PTR, posterior thalamic radiation; PTR, posterior thalamic radiation; RPIC, retrolenticular part of internal capsule; SCC, splenium of corpus callosum; SCP, superior cerebellar peduncle; SCR, superior corona radiata; SFOF, superior fronto-occipital fasciculus; SLF, superior longitudinal fasciculus; SS, sagittal stratum; UF, uncinate fasciculus.

**Fig. 8:**
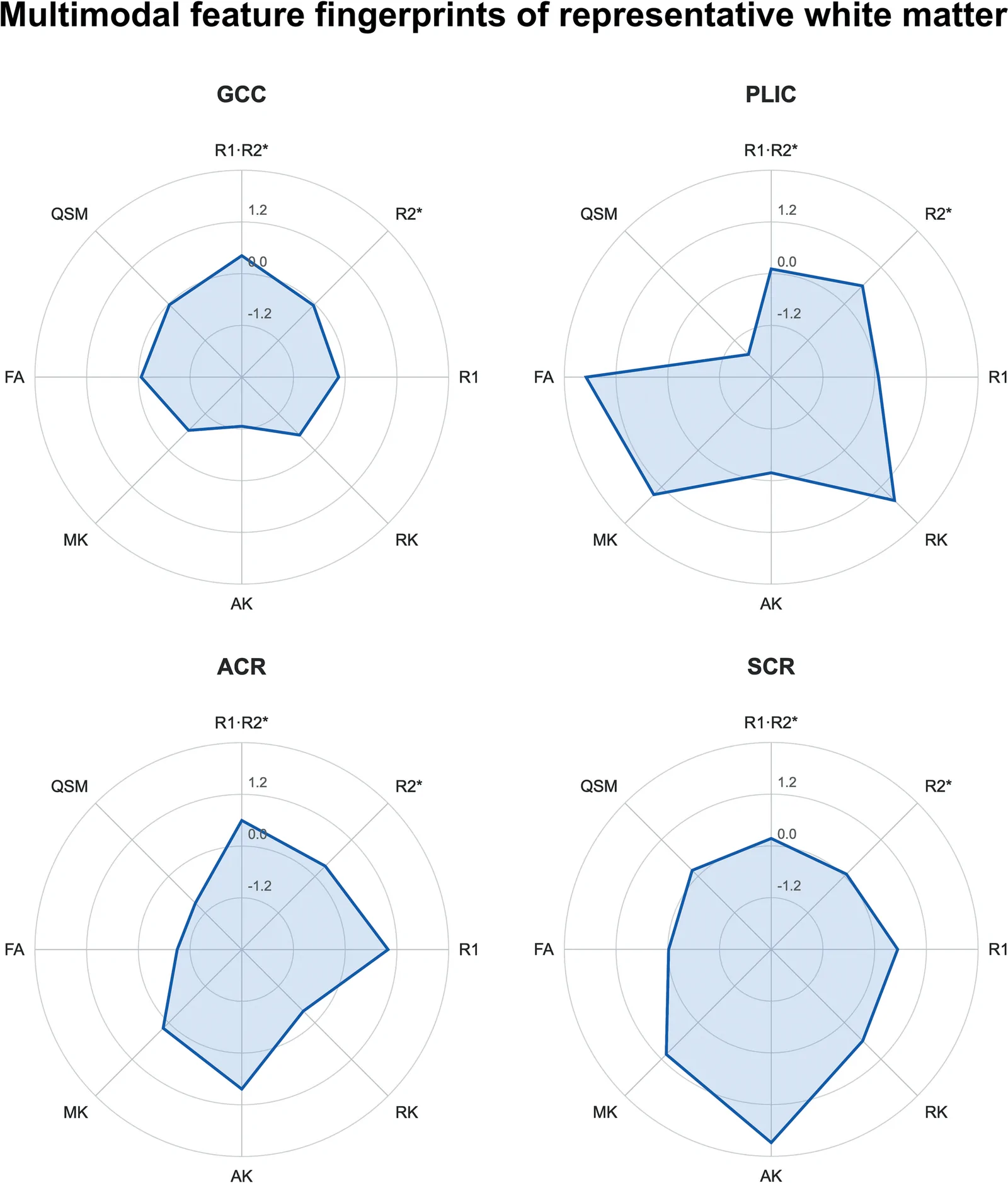
Multimodal feature fingerprints of representative bilaterally averaged white matter tracts. Radar plots illustrating standardized multimodal feature profiles for representative bilaterally averaged white matter tracts: GCC, PLIC, ACR, and SCR. Each tract exhibits a distinct combination of relaxation, susceptibility, and diffusion properties. ACR, anterior corona radiate; AK, axial kurtosis; FA, fractional anisotropy; GCC, genu of the corpus callosum; MK, mean kurtosis; PLIC, posterior limb of the internal capsule; QSM, quantitative susceptibility maps; RK, radial kurtosis; SCR, superior corona radiate.
